# Metabolic dysfunction in pregnancy: Fingerprinting the maternal metabolome using proton nuclear magnetic resonance spectroscopy

**DOI:** 10.1002/edm2.201

**Published:** 2020-11-18

**Authors:** Hannah D. Scott, Marrissa Buchan, Caylin Chadwick, Catherine J. Field, Nicole Letourneau, Tony Montina, Brenda M. Y. Leung, Gerlinde A. S. Metz

**Affiliations:** ^1^ Canadian Centre for Behavioural Neuroscience Department of Neuroscience University of Lethbridge Lethbridge AB Canada; ^2^ Department of Chemistry and Biochemistry University of Lethbridge Lethbridge AB Canada; ^3^ Department of Agriculture, Food and Nutritional Science University of Alberta Edmonton AB Canada; ^4^ Faculty of Nursing and Cumming School of Medicine University of Calgary Calgary AB Canada; ^5^ Southern Alberta Genome Sciences Centre University of Lethbridge Lethbridge AB Canada; ^6^ Public Health Program Faculty of Health Sciences University of Lethbridge Lethbridge AB Canada

**Keywords:** APrON study, biomarker, body mass index, gestational diabetes mellitus, maternal health, metabolomics, nmr spectroscopy, nuclear magnetic resonance, obesity, pathway analysis, personalized medicine, pregnancy, preterm birth, urine

## Abstract

**Aims:**

Maternal metabolic disorders place the mother at risk for negative pregnancy outcomes with potentially long‐term health impacts for the child. Metabolic syndrome, a cluster of features associated with increased risk of metabolic disorders, such as cardiovascular disease, diabetes and stroke, affects roughly one in five Canadians. Metabolomics is a relatively new technique that may be a useful tool to identify women at risk of metabolic disorders. This study set out to characterize urinary metabolic biomarkers of pregnant women with obesity and of pregnant women who later developed gestational diabetes mellitus (pre‐GDM), compared to controls.

**Methods and Materials:**

Second trimester urine samples were collected through the Alberta Pregnancy Outcomes and Nutrition (APrON) cohort and examined with ^1^H nuclear magnetic resonance (NMR) spectroscopy. Multivariate analysis was used to examine group differences, and machine learning feature selection tools identified the metabolites contributing to separation.

**Results:**

Obesity and pre‐GDM metabolomes were distinct from controls and from each other. In each comparison, the glycine, serine and threonine pathways were the most impacted. Pantothenate, formic acid and glycine were downregulated by obesity, while formic acid, dimethylamine and galactose were downregulated in pre‐GDM. The three most impacted metabolites for the comparison of obesity versus pre‐GDM groups were upregulated creatine/caffeine, downregulated sarcosine/dimethylamine and upregulated maltose/sucrose in individuals who later developed GDM.

**Conclusion:**

These findings suggest a role for urinary metabolomics in the prediction of GDM and metabolic marker identification for potential diagnostics and prognostics in women at risk.

## Graphical abstract

Urinary metabolomics can provide diagnostic signatures of obesity in pregnant mothers. The urine metabolome can predict the risk of gestational diabetes mellitus. Proton nuclear magnetic resonance spectroscopy is able to identify metabolic markers for diagnostics and prognostics in women at risk.

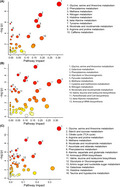

## 1 INTRODUCTION

An estimated 20% of Canadians are currently diagnosed with metabolic syndrome, a cluster of features that puts an individual at higher risk of developing diabetes and cardiovascular disease (CVD).^1^ During pregnancy, metabolic disorders such as obesity and gestational diabetes mellitus (GDM) have potentially adverse long‐term consequences for both mother and child, such as increasing the risk of preeclampsia, preterm birth, caesarean section and neonatal intensive care unit admissions.^2^, ^3^ Obesity can be a consequence of metabolic dysfunction, or a precursor to metabolic syndrome as it increases an individual's risk of developing other metabolic conditions, such as GDM or hypertension.^3^ During pregnancy, the recommended weight gain for a woman of normal weight is between 25 and 35 pounds (11‐16 kg), this amount decreases with higher pre‐pregnancy body mass index (BMI).^4^ Weight gain beyond these recommendations, or preexisting obesity, may lead to adverse pregnancy outcomes such as preeclampsia, miscarriage, congenital anomalies, preterm birth and/or foetal complications (eg, macrosomia).^5^ Furthermore, poor post‐partum weight loss serves as a predictor of future obesity.^6^

Gestational diabetes mellitus is characterized by glucose intolerance with onset or first recognition during pregnancy, and it affects an estimated 3.7% of all pregnancies in Alberta.^7^ Despite its prevalence, the initial pathogenic mechanisms of GDM are not fully understood. While GDM is strongly associated with the development of type 2 diabetes later in life, many individuals diagnosed with GDM have no prior known metabolic dysfunction.^8^ Inter‐ and transgenerational inheritance of GDM risk, however, has previously been suggested.^9^, ^10^ While both GDM and obesity are individually associated with adverse pregnancy outcomes, in conjunction they have synergistic effects. Hence, the 2012 Hyperglycemia and Adverse Pregnancy Outcome (HAPO) study reported that mothers with both GDM and obesity had significantly increased birth weight, newborn body fat, caesarean delivery rates and prevalence of preeclampsia when compared to individuals with only one, or neither, risk factor.^11^

Despite the increased risk of the said adverse health outcomes associated with metabolic syndrome, obesity or GDM, not all individuals with either condition go on to develop these disorders. The underlying mechanisms that cause conditions to worsen in some individuals but not others are not fully understood; however, cross‐sectional analysis of at‐risk individuals may address this gap by identifying factors of risk that have not yet been considered.^12^, ^13^, ^14^ In many cases, such as prediabetes, mitigation of these factors can improve health outcomes, and even prevent the disease state.^13^, ^15^ Metabolomics, the study of the metabolism and associated pathways, presents an effective tool for biomarker discovery to identify high‐risk individuals for targeted interventions and disease prevention.^16^, ^17^ A recent study investigating the early pregnancy serum metabolomic profile of overweight and obese women identified several biomarkers that predict risk of developing GDM,these included small high‐density lipid (HDL) particles, branched chain amino acids (BCAAs) and inflammatory markers.^18^

The present study aims to identify robust biomarkers associated with obesity and GDM status in the urinary metabolome. Urinary metabolomics enables the noninvasive detection of metabolic diseases during pregnancy without the need for a blood test. This study uses ^1^H nuclear magnetic resonance (NMR) spectroscopy to investigate metabolomic signatures linked to obesity and GDM in the urine of pregnant women collected in the second trimester from the Alberta Pregnancy Outcomes and Nutrition (APrON) study.^19^ We hypothesized that women with obesity or GDM will show characteristic urinary metabolomic signatures that differ from controls.

## 2 MATERIALS AND METHODS

### 2.1 Study design

Urine samples were collected from pregnant women in the Alberta Pregnancy Outcomes and Nutrition (APrON) study, a Canadian pregnancy cohort study.^20^ The APrON study was created to examine the links between perinatal nutrition intake and birth outcomes, child development, and maternal mental health.^19^ The full cohort consists of 2140 women, 2172 infants and 1417 biological fathers recruited in Calgary (population 1.1 million) and Edmonton (population 0.9 million), Alberta, Canada. The methodology details for the APrON study have been published elsewhere.^19^, ^20^

The present study used urine samples collected between 14 and 27 weeks of pregnancy from 29 women with obesity, 37 with GDM and 36 healthy controls. The three groups were identified as follows: (a) pregnant women (nondiabetic) with obesity were classified according to BMI status >30, or waist circumference >30); (b) pregnant women with GDM were extracted from hospital records whose diagnosis of GDM came from routine prenatal screening for GDM according to the Canadian Diabetes Association criteria; (c) controls were pregnant women without obesity or diabetes, matched for age, family income, education. Pregnant women were screened for GDM according to the Canadian Diabetes Association criteria of a 1‐hour plasma glucose (1hPG) measurement following a 50‐gram glucose load given at any time of day. If the 1hPG was ≥10.3 mmol/L, or if it was >7.9 mmol/L and a subsequent 2 hour 75 g glucose load was positive, GDM was confirmed. Screening for GDM is part of routine prenatal care at ~24‐28 weeks gestation. The GDM cases were identified based on hospital records. Each case of obesity or GDM was counted only once. Women with obesity did not go on to develop GDM in the selected cases. Nine of the 37 GDM cases (23.7%) had a pre‐pregnancy BMI >30. Controls were matched by age‐, income‐ and education level. Table 1 provides the characteristics of the participants in this study.

**TABLE 1:** Characteristics of the participants in this study

Participant Information	Control (n = 36)	GDM (n = 37)	Obese (n = 29)
Age			
Mean (standard deviation)	33.4 (4.5)	33.8 (3.9)	32.8 (4.2)
Pre‐pregnancy BMI			
Mean (standard deviation )	23.1 (2.2)	27.7 (6.5)	33.2 (4.0)
Income			
$20 000‐$39 999	2	3	3
$40 000‐$69 999	9	7	4
$70 000‐$99 999	11	11	11
$100 000 or More	14	16	11
Education			
Completed High School Diploma	3	5	3
Completed Trade, Technical	5	6	4
Completed University	16	15	13
Completed Post‐Grad	12	11	9
Marital status			
Single	0	0	2
Married	35	36	26
Common‐Law	1	0	1
Unknown		1	
Ethnicity			
Caucasian	28	28	26
Other	8	9	3

### 2.2 Sample collection and preparation

Urine samples were obtained midstream during the first passage of the day following an overnight fasting period. The samples were stored at −80°C. Samples were thawed at room temperature, and 400 μL of urine was added to 200 μL of phosphate buffer containing a 4:1 ratio of dibasic potassium phosphate (K_2_HPO_4_) to monobasic potassium phosphate (KH_2_PO_4_) with a combined concentration of 0.5 M (pH 7.4) in 80% H_2_O and 20% D_2_O. The D_2_O contained 0.03% (w/v) 3‐(trimethylsilyl)propionic acid (TSP) to be used as an internal chemical shift reference and quantification standard. Three mmol/L of sodium azide (NaN_3_) were added as anti‐microbial agent. The urine/buffer mixture was gently vortexed until homogenous and then centrifuged at 10 600 *g* for 5 minutes at 4°C. 550 μL of supernatant was pipetted into 5 mm NMR tubes before proceeding with ^1^H‐NMR spectroscopy.

### 2.3 NMR data acquisition and processing

Nuclear magnetic resonance spectra of the urine samples were acquired at room temperature using a 700 MHz Bruker Avance III HD NMR Spectrometer (Bruker) equipped with a 5 mm triple resonance TBO‐Z probe. The one‐dimensional NOESY gradient water suppression pulse sequence (noesygppr1d) was utilized with the following parameters: mixing time of 10 ms; 128 k data points (TD); sweet width (SW) of 20.52 ppm, acquisition time (AQ) of 4.56 seconds, transmitter offset (o1p) of 4.6 ppm; recycle delay (D1) of 1 second; 128 scans (NS). Spectra were then processed using zero‐filling to 256 k points, line broadening with a 0.3 Hz exponential multiplication, automatic phased and baseline‐correction, and chemical shift referenced with respect to the TSP peak at 0 ppm. All spectra were converted to ascii files and exported to MATLAB (MathWorks) for further analysis. The spectra underwent dynamic adaptive binning,^21^ followed by manual adjustment to correct for any errors in the algorithm. The bins containing the water and urea peaks were removed resulting in a total of 277 bins for all urine spectra. The spectra were normalized to the total area of all bins (the total metabolome), log transformed and Pareto scaled.^22^

### 2.4 Statistical analyses

For each comparison (obese vs control; GDM vs control; obese vs GDM), bins underwent both univariate and multivariate testing. Univariate testing provides a statistical measure by which each of the spectral bins can be tested to determine whether it has been significantly altered across the comparison groups on an individual basis. Multivariate testing offers a method by which each of the bins can be statistically assessed with respect to their importance to class separation when considered as part of a complete set of variables. Thus, univariate and multivariate testing provide complementary information about the importance of a bin, or metabolite, to observed group differences.

#### 2.4.1 Univariate testing

The decision tree algorithm outlined by Goodpaster et al^23^ was utilized to ensure that the appropriate univariate test was applied to the bins. This decision tree algorithm first uses a Shapiro‐Wilk test to determine whether the bins are normally distributed. If the bins follow a non‐normal distribution, they undergo a Mann‐Whitney *U* test (MW) for significance. In the case of this study, the data were determined to follow non‐normal distributions and the Mann‐Whitney *U* test was applied.

#### 2.4.2 Multivariate testing

Variable Importance Analysis based on random Variable Combination (VIAVC)^24^ was utilized to assess variable significance when considered as part of the total set of variables. The VIAVC algorithm utilizes binary matrix resampling to create several random subsets of variables containing 50% of the original bins. In the case of this study, 1000 subsets of random variables were used. Binary matrix resampling ensures that each variable has been selected with the same probability when creating the random subsets. The importance of each bin is then assessed by calculating the corresponding area under the curve (AUC) for the receiver operator characteristic (ROC) curve with and without a variable included in the model. If the inclusion of the variable increases or decreases the area under the curve, it is either retained or removed from the overall data set, respectively. This process is repeated until no more variables can be removed and the resulting list of bins is known as the best subset. By doing this, the VIAVC algorithm can determine if any synergistic effects exist between variables that appear nonsignificant based on traditional univariate and multivariate measures. In addition, it can remove bins that are significant based on a univariate test but reduce group seperation when they are included in the whole set of variables. This machine learning approach is an ideal method for determining the subset of variables which lead to the best separation between two classes or groups.

Metaboanalyst^25^, ^26^ was utilized to carry out and visualize both the orthogonal partial least squares discriminant analysis (OPLS‐DA) and receiver operating characteristic (ROC) curves. The former was carried out using bins identified as significant by the MW or VIAVC tests. The latter was performed using only the bins identified as significant by the VIAVC test. All modelling underwent permutation testing (2000 permutations) and double ten‐fold cross‐validation.^27^

#### 2.4.3 Metabolite identification and pathway analysis

Both Chenomx (Chenomx) and the Human Metabolome Database (HMDB)^28^, ^29^, ^30^, ^31^ were used to identify the metabolites present in each of the significant bins. The complete list of metabolites identified as significantly altered by either the MW test or VIAVC algorithm were used for pathway topology analysis.^32^ Pathway topology analysis was carried out in Metaboanalyst by selecting the hypergeometric test for the over‐representation analysis, relative‐betweenness centrality for the topology analysis, and using the Kyoto Encyclopedia of Genes and Genomes (KEGG) database for *Homo sapiens* ^33^, ^34^, ^35^ as the pathway library.

## 3 RESULTS

**FIGURE 1:**
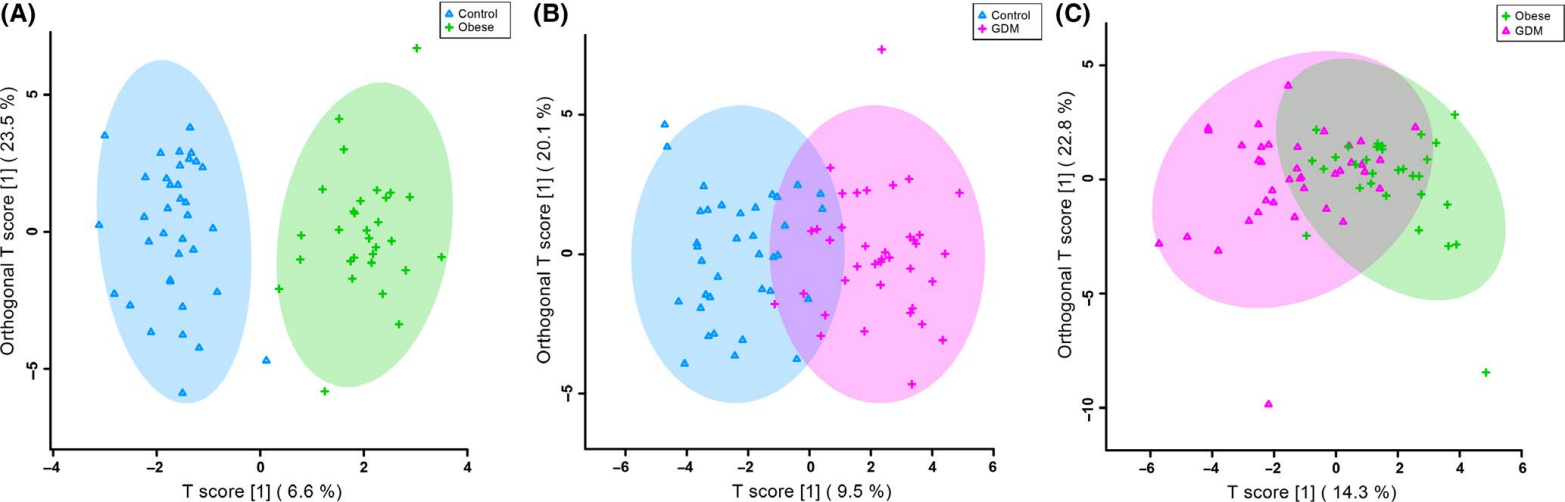
Orthogonal partial least squares discriminant analysis (OPLS‐DA) score plots showing supervised group seperation between (A) obesity and controls (R^2^X = 0.89, Q^2^ = 0.574, R2Y *P*‐value = .0005, Q2 *P*‐value = .0005), (B) GDM and controls (R^2^X = 0.722, Q^2^ = 0.277, R2Y *P*‐value = .0005, Q2 *P*‐value = .0005), and (C) obesity and GDM (R^2^X = 0.465, Q^2^ = 0.258, R2Y *P*‐value = .0005, Q2 *P*‐value = .0005). Each triangle or cross represents one individual under study, plotted using a list of urinary metabolites found to be statistically significant by either MW or VIAVC testing. The *x*‐axis and *y*‐axis show the predictive (across group variation) and orthogonal (within group) components, respectively

**FIGURE 2:**
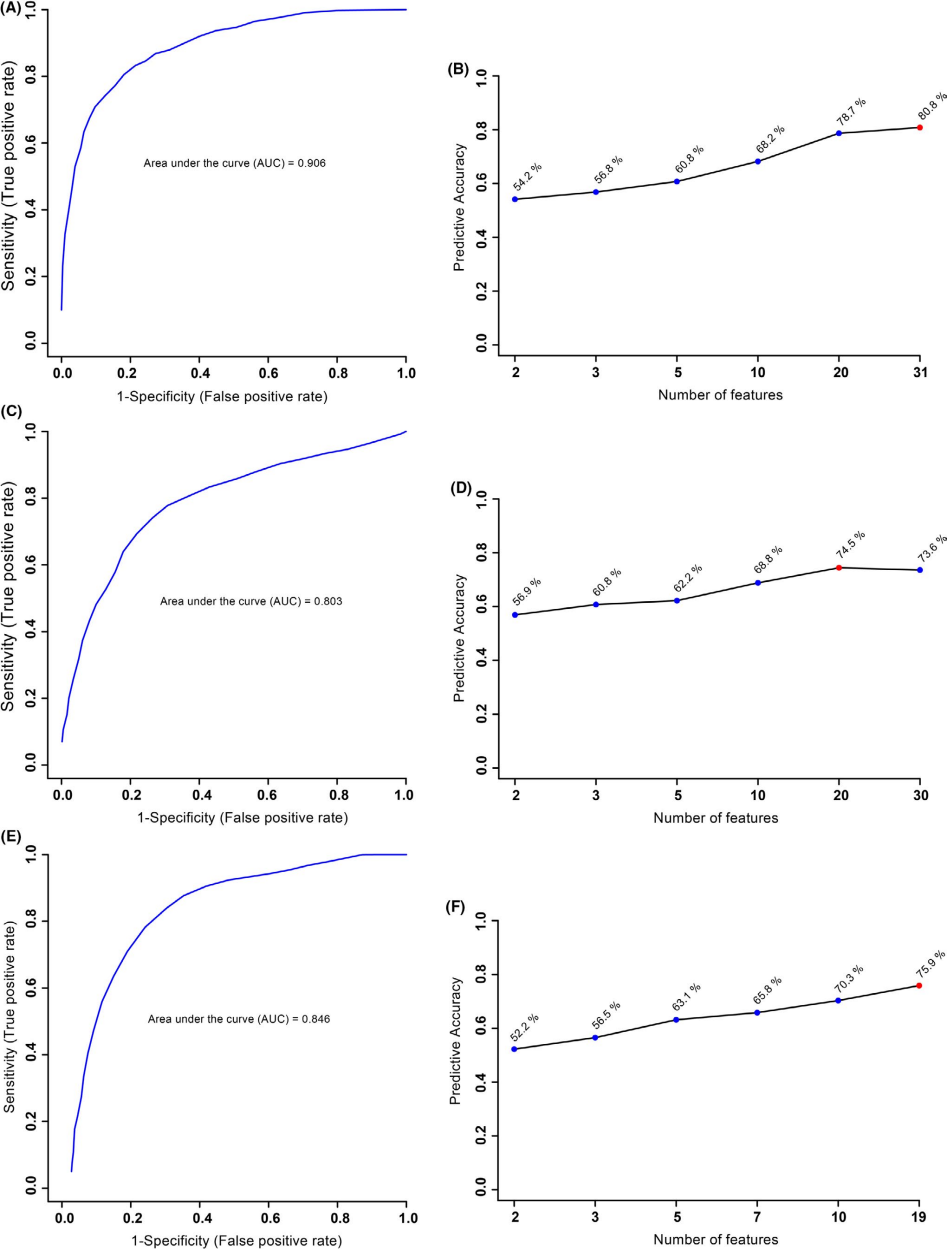
Receiver operating characteristic (ROC) curve (A, C, E) and predictive accuracy (B, D, F) for the comparison of (A, B) obesity and controls, (C, D) GDM and controls, and (E, F) obesity and GDM. These figures were created using the best subset of metabolites as determined by the VIAVC analysis. The 95% confidence intervals for A, C and E are 0.772‐0.986, 0.655‐0.942 and 0.603‐0.962, respectively

Of the 277 total spectral bins, both VIAVC and MW tests identified which bins led to significant group separation. Analyses were applied to three comparison groups and resulted in the following number of significant bins: obesity vs. control (33 MW, 31 VIAVC and nine common bins); GDM vs control (61 MW, 30 VIAVC and 12 common bins); and obesity vs GDM (30 MW, 19 VIAVC and eight common bin). The supervised OPLS‐DA comparisons of the obese and control (Figure 1A), GDM and control (Figure 1B), and GDM and obesity (Figure 1C) all showed significant group separation with the largest separation between the obese and control groups. Permutation and cross‐validation tests confirmed this observed separation for the three comparisons (*P* < .05). Receiver operator characteristic (ROC) curves were used to determine the specificity, sensitivity and accuracy of each comparison model based on metabolites identified as significant by VIAVC. The comparisons between obesity and control, GDM and control, and GDM and obesity gave an area under the curve (AUC) and predictive accuracy (in brackets) of 0.906 (80.8%), 0.803 (74.5%) and 0.846 (75.9%), respectively (Figure 2).

Tables [Link], [Link], [Link] provide the metabolites that were found to be significantly altered by either Mann‐Whitney *U* or VIAVC. The last column on each table indicates whether the metabolite was up‐ or downregulated. A total of 58 metabolite bins significantly contributed to the distinction between the obesity vs control group, 79 GDM vs control group and 41 to the obesity vs GDM comparison. A total of 50 out of 58 and 65 out of 79 significantly altered bins were downregulated compared to the controls in both the obesity and GDM groups, respectively. No such trend was observed in the obesity vs GDM comparison.

The most impacted three metabolites for the comparison of obesity and control groups were pantothenate, formic acid and glycine with all three metabolites downregulated in the obesity group. The top three metabolites for the comparison of GMD and control groups were formic acid, dimethylamine and galactose with all three metabolites downregulated in the GDM group. The top three metabolites for the comparison of GDM and obesity groups were creatine/caffeine, sarcosine/dimethylamine and maltose/sucrose.

**FIGURE 3:**
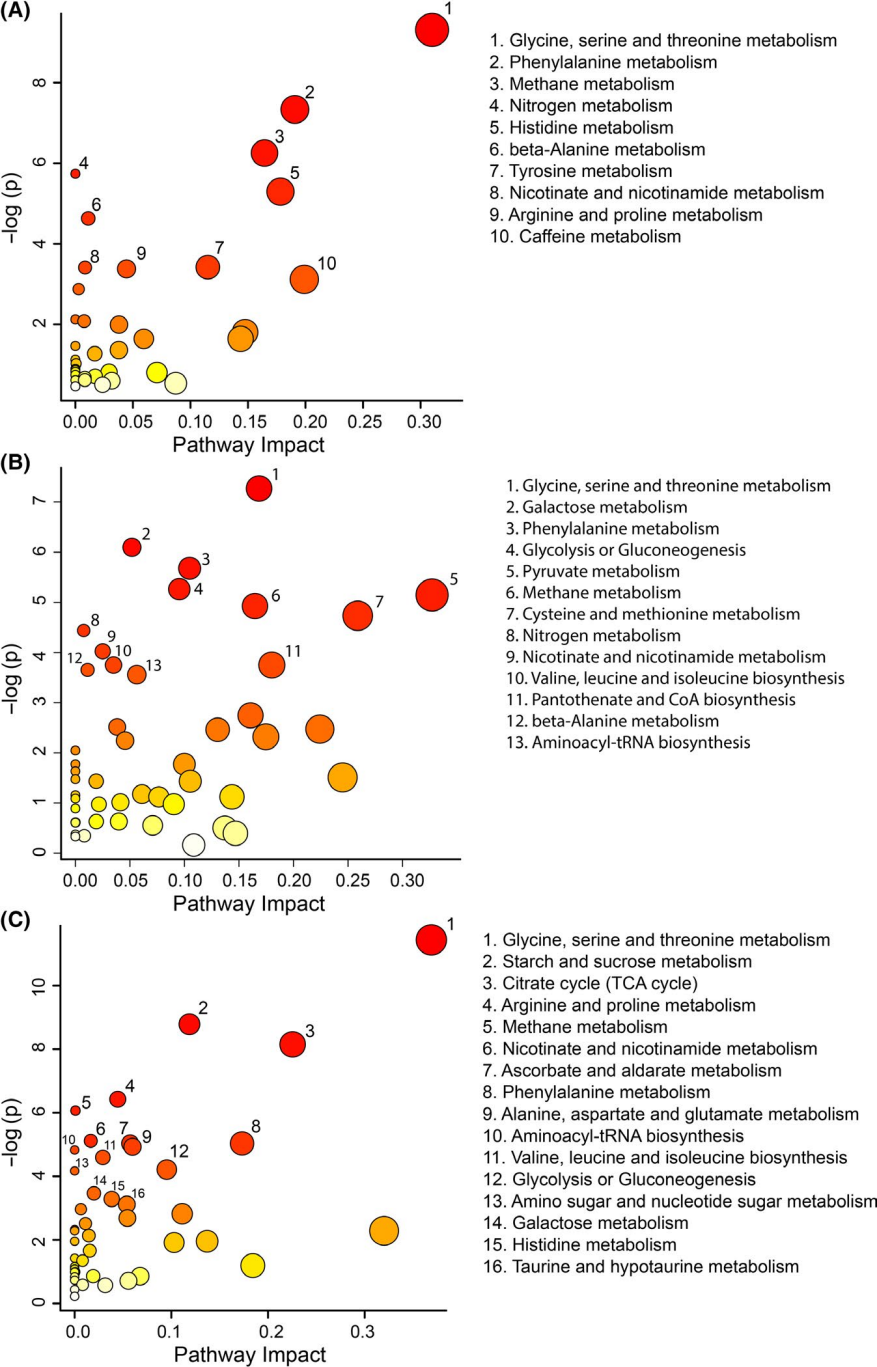
Metabolomic pathway analysis for the comparison of the obesity vs control groups (A), GDM vs control groups (B), and obesity vs GDM groups (C). A higher value on the y‐axis indicates a lower *P*‐value for the pathway. The x‐axis gives the pathway impact, which indicates how affected the pathway is by the metabolites identified as significantly altered. Only metabolic pathways with *P* < .05 are labelled. This figure was created using the lists of metabolites identified as significantly altered between the obese and control groups by either MW or VIAVC testing

For the obese and control comparison, the metabolomic pathway analysis identified 10 significantly altered pathways (Figure 3A), with glycine, serine and threonine metabolism (*P* < .05), phenylalanine metabolism (*P* < .05), and methane metabolism (*P* < .05) being the most significantly altered. In the GDM and control comparison, 13 significantly altered pathways were identified (Figure 3B), including glycine, serine and threonine metabolism (*P* < .05), galactose metabolism (*P* < .05), and phenylalanine metabolism (*P* < .05). In the obesity and GDM comparison, 16 metabolic pathways were significantly altered (Figure 3C), with the greatest impact on glycine, serine and threonine metabolism (*P* < .05), starch and sucrose metabolism (*P* < .05), and the citrate cycle (*P* < .05).

## 4 DISCUSSION

This study demonstrated that ^1^H‐NMR metabolomics can be used to distinguish and compare the urinary metabolomes of healthy pregnant woman to those diagnosed with obesity and may be predictive of women who develop GDM. Metabolites or impacted pathways found to be distinct between groups may serve as indicators of risk, allowing for the use of early clinical or lifestyle intervention prior to manifestation of a disease state.^36^ Characterization of the metabolic processes altered by obesity and GDM makes an important contribution to understanding their pathogenic mechanisms.^8^ The present results provide insight into the comorbidity existing between obesity and GDM. The similarity of the metabolomes between these disease states indicates shared biochemical pathway disruptions which may be detectable in individuals prior to the development of associated adverse health outcomes.

The high predictive accuracy of the three ROC curves resulting from this study (80.8% for obesity vs control, 74.5% for GDM vs control and 75.9% for obesity vs GDM) demonstrates that the best subset of metabolites as determined by the VIAVC analysis can be used to distinguish between the groups. While the R^2^X values for the GDM vs control comparisons and obesity vs control indicated a large degree of variability (>0.7), the obesity vs GDM comparison was 0.465, demonstrating only a moderate difference between the groups. This indicates the presence of similar underlying mechanisms and metabolic disturbances shared between obesity and GDM. No single metabolite or pathway was responsible for the majority of this separation, which was not surprising considering the multifaceted pathogenic mechanisms underlying obesity and GDM. While the best subset of metabolites provides the most accurate diagnostic tool, examining the metabolites and pathways individually provides insight into the underlying mechanisms of obesity and GDM, and can help provide a better understanding of the risk factors behind each condition.

While multiple metabolic pathways were impacted by the presence of obesity and GDM, altered glycine, serine and threonine metabolism pathway function was among the most distinct signatures for both conditions. In this pathway, serine is derived from glycolysis and in turn is converted into glycine. Threonine is an essential amino acid derived from diet, which is also converted into glycine.^37^ Glycine deficiency in particular has been found to be associated with increased abdominal adipose tissue,^38^ potentially contributing to the downregulation of the metabolite observed in the obese group.

The phenylalanine metabolism was found in the top three significantly impacted pathways when comparing both obesity and GDM to control groups. Phenylalanine is an aromatic amino acid that acts as a precursor to tyrosine, along with multiple catecholamines including epinephrine, norepinephrine and dopamine. Metabolic disorders such as obesity and GDM have been found to lead to elevations in phenylalanine and several of its metabolic products.^39^ Elevated levels of aromatic amino acids have been associated with obesity^12^ and the development of insulin resistance in nondiabetic individuals.^40^ BCAAs, particularly valine, leucine and isoleucine, are often presented as indicators of risk for insulin resistance alongside aromatic amino acids^41^, ^42^ and have been implicated in the development of GDM in overweight and obese pregnant women.^18^ In this study's comparison between individuals with obesity and GDM, the BCAA leucine was significantly upregulated in the GDM group alongside phenylalanine (Table S3), and valine was found to be higher in individuals with GDM versus their control counterparts (Table S2), supporting the role of BCAAs in the development of insulin resistance. Thus, phenylalanine, leucine and valine provide valuable urinary biomarkers of GDM risk in obese individuals.

It should be noted that caution must be taken when comparing studies using different biofluids, as metabolite expression can depend on a variety of cohort factors such as circadian rhythms, diet and physical activity. In the present study, the downregulation of galactose in individuals with GDM seemingly conflicts with previous findings that serum d‐galactose is upregulated in response to the disease.^43^ In addition, pantothenate, the most impacted individual metabolite in the obesity vs control comparison, represents another case of discrepancy between serum and urinary expression. While the present downregulation of urinary pantothenate may serve as a urinary indicator of obesity, serum pantothenate has been found to be upregulated in response to obesity.^44^ As mentioned above, these discrepancies may be due to differences between the study cohorts. It cannot be ruled out, however, that excreted galactose and pantothenate in the urine may reflect serum levels. For example, a metabolite being upregulated in blood may indicate increased demand for the metabolite, which would result in reduced excretion of the metabolite in the urine. As mentioned earlier, urinary metabolomics enables the noninvasive detection of metabolites during pregnancy without the need for a blood test. In addition, NMR‐based urinary metabolomics provides information on 209 metabolites compared to only 49 metabolites via serum metabolomics.^45^, ^46^

The regulation of sucrose expression appears to also vary between the serum and urinary metabolomes. Increased intake of sucrose has been linked to insulin resistance in mice.^47^ However, both the obese and GDM groups of this study experienced reduced urinary sucrose levels when compared to control individuals. This may be partially explained by sex differences,sucrose‐induced insulin‐resistant models have only been successfully created in male animals, and females appear to be resistant to sucrose‐induced insulin resistance.^48^ When comparing individuals with obesity to those with GDM, sucrose was the third most impacted metabolite, being increased in GDM individuals. This finding indicates that starch and sucrose metabolism does indeed affect females and potentially contributes to the development of GDM, which highlights the need for a better understanding of sex differences in diabetes‐related health outcomes, such as coronary heart disease.^49^

The most impacted pathway distinction between obesity and GDM concerned starch and sucrose metabolism. High starch diets appear to have the opposite effect of sucrose on insulin resistance, reducing its severity and decreasing adipose tissue weight.^50^ Variations in starch and sucrose metabolism may reflect dietary variations in the subjects, which in turn may correlate with the presence of obesity and GDM states.^51^ It should also be noted that, while this study controlled for age, income and education level, subjects underwent no dietary restrictions prior to sampling, so impacts of diet on the metabolome act as a potential confound.

The results of this study allow for further analysis of the comorbidity of obesity and GDM with their associated health risks. Preeclampsia is one of the most severe pregnancy complications associated with GDM. The high blood pressure of this complication results from the improper formation of blood vessels in the placenta, and can cause organ damage, and even maternal and foetal death if not addressed. The presence of GDM more than doubles the risk of a mother developing preeclampsia.^52^ Several of the most impacted urinary metabolites in the GDM vs control (formic acid, ethanol and propylene glycol) and obesity vs control (formic acid and glycine) comparisons of this study are known to be associated with alterations to the serum metabolome found in early preeclampsia^53^ and may be indicative of shared pathway disruptions between the conditions.

Spontaneous preterm birth, when the infant is delivered prior to 37 weeks of gestation, is another pregnancy complication that is associated with metabolic syndrome, obesity and GDM obesity.^2^, ^54^, ^55^ Previous urinary metabolic analysis of preterm birth revealed an association between spontaneous preterm birth and decreased levels of formic acid.^56^ This trend was also observed in the obese and GDM groups of this study when compared to control individuals. The mechanisms by which this trend is associated with preterm birth are not yet known, but it was suggested that diminished urinary formic acid raises the risk of hypertension,^57^ which in turn is positively associated with preterm birth.^2^

### 4.1 Synthesis and conclusion

This exploratory study contributes to the understanding of the biochemical mechanisms of both obesity and GDM and supports the role of ^1^H NMR spectroscopy metabolomics in the developing field of precision medicine. The individual metabolites that contributed most to separation between disease groups have the highest potential to provide simple diagnostic tests. The creation of new tests for precision medicine approaches for intervention necessitates a deeper understanding of the underlying metabolic mechanisms behind obesity, GDM and pregnancy. The identified pathways provide insight into the underlying mechanisms of obesity and GDM and potential therapeutic targets.

While the present findings suggest a composite metabolic profile to be the most robust predictor, the findings also indicate that the glycine, serine and threonine metabolism pathway may provide the most feasible potential to provide a single component biomarker to distinguish obesity and GDM from healthy individuals. Moreover, the starch and sucrose metabolism pathway provides the most distinction between the urinary metabolome of individuals with GDM and those who are obese. The upregulation of BCAA and aromatic amino acids also provides an effective biomarker for the development of GDM in obese individuals and suggests possible causal mechanisms for insulin resistance. This study also supports further investigation of the urinary metabolome as a noninvasive diagnostic tool, as it delivers powerful results without the need for a serum sample. Using biomarkers to determine and potentially prevent metabolic disease ultimately may not only improve quality of life for mothers and their children, but also assist in the transition to preventative and precision medicine.

## CONFLICT OF INTEREST

The authors have no conflicts or competing interests to declare.

## AUTHOR CONTRIBUTION

B.L., C.F. and N.L. led the cohort and participant recruitment. G.M. and T.M. designed the metabolomics portion of the study. H.S., M.B. and C.C. analysed the samples and performed data analysis. H.S., C.C., T.M., B.L. and G.M. prepared the manuscript. All authors have approved the final version of this manuscript.

## Supporting information

Table S1

Table S2

Table S3

## Data Availability

The data that support the findings of this study are available from the corresponding author upon reasonable request.
