# Supplementary material for: Metabolic dysfunction in pregnancy: Fingerprinting the maternal metabolome using proton nuclear magnetic resonance spectroscopy
Source: Endocrinol Diabetes Metab. 2020 Nov 18;4(1):e00201. doi: 10.1002/edm2.201 (PMC7831222; doi:10.1002/edm2.201)
Supplement: Supplementary file 1 — Table S1 [file EDM2-4-e00201-s001.docx]

**Supplementary Table 1.** P-values of urinary metabolites found to be significant in separation of obese and control groups in either a Mann-Whitney U test, the Variable Importance Analysis based on random Variable Combination (VIAVC), or both. Regulation is shown for the metabolite levels in the obese group. Metabolites for which more than one NMR resonance peak was identified as significant are represented as metabolite.1, metabolite.2, … metabolite.n.

| Metabolite | Mann Whitney U Test | VIAVC  p-value | Regulation |
| --- | --- | --- | --- |
| *Pantothenate.1* | 1.02E-02 | 3.51E-149 | Down |
| *Formic acid* | 2.64E-03 | 6.48E-116 | Down |
| *Glycine* | - | 1.64E-110 | Down |
| *Caffeine.2, Methanol* | 6.71E-03 | 1.34E-106 | Down |
| *3,4-Dihydroxybenzeneacetate* | - | 7.39E-103 | Up |
| *Sarcosine, Dimethylamine* | - | 1.49E-67 | Down |
| *3-Hydroxyphenylacetic acid* | - | 5.08E-64 | Down |
| *Anserine.2* | 1.52E-02 | 7.72E-63 | Down |
| *N,N-Dimethylformamide.2, 3-Phenylpropionate* | - | 3.63E-56 | Up |
| *Unidentified metabolite (Doublet at 6.488ppm)* | 3.12E-03 | 2.96E-46 | Down |
| *Glucuronic acid* | - | 8.00E-46 | Down |
| *m-Thymol* | 6.21E-03 | 1.78E-41 | Down |
| *2-Octenoate.2, Caprate, 2-Hydroxyisovalerate* | - | 1.92E-37 | Down |
| *Fucose.2* | - | 7.35E-33 | Up |
| *Fucose.1* | - | 5.05E-25 | Up |
| *N-Acetylaspartate.3* | - | 1.32E-23 | Down |
| *Homoserine* | - | 8.99E-21 | Down |
| *Methylsuccinic acid, Isobutyric acid.2* | - | 8.91E-17 | Up |
| *Fumaric acid* | 1.69E-02 | 4.23E-15 | Down |
| *5-Aminolevulinate* | - | 1.90E-14 | Up |
| *N,N-Dimethylformamide.1* | - | 6.39E-11 | Down |
| *Sucrose.2* | 2.46E-02 | 4.37E-09 | Down |
| *N-Acetylglutamine* | - | 2.97E-08 | Down |
| *3-Hydroxymandelic acid* | - | 1.53E-07 | Down |
| *2-Methylglutaric acid, 3-Hydroxyisobutyric acid, Isobutyric acid.1, 3-Methyl-2-oxovaleric acid* | - | 2.32E-07 | Down |
| *Caffeine.1, 3-Methylxanthine* | - | 6.63E-06 | Down |
| *Chlorogenate* | - | 9.35E-06 | Up |
| *Malonate* | - | 1.09E-04 | Down |
| *Cholate* | - | 2.87E-04 | Down |
| *Unidentified metabolite (Peak at 6.502ppm)* | 2.75E-03 | 6.51E-03 | Down |
| *Creatinine* | - | 1.34E-02 | Up |
| *1,3-Dihydroxyacetone* | 3.75E-02 | - | Down |
| *2-Hydroxybutyric acid, Histidine, Phenylalanine.1* | 1.81E-02 | - | Down |
| *2-Octenoate.1* | 3.10E-02 | - | Down |
| *ADP, Anserine.1* | 3.20E-02 | - | Down |
| *Ascorbic acid* | 1.14E-02 | - | Down |
| *Cinnamic acid.1* | 8.14E-03 | - | Down |
| *Cinnamic acid.2* | 4.00E-02 | - | Down |
| *Galactose* | 9.82E-03 | - | Down |
| *Hippuric acid, 2-Furoylglycine, Creatine* | 4.00E-02 | - | Down |
| *Histamine, 1-Methylhistidine, Caffeine.3* | 3.39E-03 | - | Down |
| *Homogentisic acid.1* | 6.98E-03 | - | Down |
| *Homogentisic acid.2* | 4.25E-02 | - | Down |
| *Hydroxyacetone* | 2.72E-02 | - | Down |
| *Maleic acid* | 4.25E-02 | - | Down |
| *N-Acetylaspartate.1* | 1.69E-02 | - | Down |
| *N-Acetylaspartate.2* | 3.10E-02 | - | Down |
| *Pantothenate.2* | 4.95E-02 | - | Down |
| *Phenylalanine.2* | 1.52E-02 | - | Down |
| *Sucrose.1* | 3.87E-02 | - | Down |
| *Tartrate* | 4.25E-02 | - | Down |
| *Trigonelline.1* | 2.91E-02 | - | Down |
| *Trigonelline.2* | 3.64E-02 | - | Down |
| *Trigonelline.3* | 3.64E-02 | - | Down |
| *Trigonelline.4* | 3.87E-02 | - | Down |
