# Supplementary material for: Metabolic dysfunction in pregnancy: Fingerprinting the maternal metabolome using proton nuclear magnetic resonance spectroscopy
Source: Endocrinol Diabetes Metab. 2020 Nov 18;4(1):e00201. doi: 10.1002/edm2.201 (PMC7831222; doi:10.1002/edm2.201)
Supplement: Supplementary file 2 — Table S2 [file EDM2-4-e00201-s002.docx]

**Supplementary Table 2.** P-values of urinary metabolites found to be significant in separation of GDM and control groups in either a Mann-Whitney U test, the Variable Importance Analysis based on random Variable Combination (VIAVC), or both. Regulation is shown for the metabolite levels in the GDM group. Metabolites for which more than one NMR resonance peak was identified as significant are represented as metabolite.1, metabolite.2, … metabolite.n.

| Metabolite | Mann Whitney U Test | VIAVC  p-value | Regulation |
| --- | --- | --- | --- |
| *Formic acid* | 8.82E-04 | 1.21E-173 | Down |
| *Dimethylamine* | 2.21E-04 | 2.79E-111 | Down |
| *Galactose* | 9.01E-03 | 6.23E-84 | Down |
| *Anserine.3* | 2.62E-04 | 3.15E-75 | Down |
| *2,3,4-Trihydroxybenzoic acid.2* | 2.69E-03 | 3.37E-65 | Down |
| *N-Methylhydantoin, 5-Aminolevulinic acid.2* |  | 1.06E-63 | Up |
| *1,3-Dimethyluric acid* | 1.02E-02 | 4.72E-58 | Down |
| *3-Hydroxymandelic acid* | 5.01E-03 | 1.39E-52 | Down |
| *Succinylacetone* | - | 3.46E-51 | Up |
| *Propylene glycol* | - | 8.04E-48 | Up |
| *5-Aminolevulinic acid.1* | - | 6.71E-40 | Up |
| *Caffeine.1* | - | 1.50E-36 | Down |
| *3-Phenylpropionic acid.2* |  | 1.55E-34 | Down |
| *2-Phenylpropionic acid* | - | 1.07E-28 | Down |
| *Trigonelline.1* | - | 3.22E-27 | Down |
| *Lactose.1* | - | 6.25E-26 | Up |
| *Theophylline* | - | 7.06E-23 | Down |
| *Trigonelline.2* | - | 8.72E-23 | Down |
| *Desaminotyrosine, N-Acetyltyrosine, 4-Hydroxyphenylacetic acid* | 7.68E-03 | 2.14E-18 | Down |
| *Homogentisic acid.2* | 6.97E-03 | 6.03E-18 | Down |
| *Methanol* | - | 2.07E-17 | Down |
| *Valine* | - | 9.05E-14 | Up |
| *Pyruvic acid* | - | 1.00E-13 | Down |
| *Pantothenic acid* | 4.15E-02 | 3.87E-12 | Down |
| *Lysine* | - | 8.17E-12 | Down |
| *Asparagine* | - | 1.27E-11 | Up |
| *Mannose* | 5.72E-03 | 2.61E-09 | Down |
| *3,4-Dihydroxybenzeneacetic acid* | - | 1.86E-08 | Up |
| *S-Adenosylhomocysteine, 2'-Deoxyadenosine* | 5.01E-03 | 4.51E-08 | Down |
| *Ascorbic acid* | - | 9.17E-07 | Up |
| *ADP, Anserine.1* | 2.63E-02 |  | Down |
| *Anserine.2* | 4.48E-02 |  | Down |
| *Caffeine.2, 1-Methylhistidine, Histamine* | 1.02E-02 |  | Down |
| *Indoxyl sulfate.2, Tryptophan* | 4.53E-03 |  | Down |
| *Indoxyl sulfate.1* | 4.15E-02 |  | Down |
| *Protocatechuic acid* | 9.59E-03 |  | Down |
| *Thymol* | 5.07E-04 |  | Down |
| *Homogentisic acid.1* | 4.48E-02 |  | Down |
| *2-Octenoic acid.1* | 3.18E-02 |  | Down |
| *2-Octenoic acid.2* | 1.05E-02 |  | Down |
| *2-Octenoic acid.3* | 2.55E-02 |  | Down |
| *2,3,4-Trihydroxybenzoic acid.1* | 3.36E-02 |  | Down |
| *Maleic acid, Allantoin* | 5.01E-03 |  | Down |
| *Levoglucosan* | 4.04E-02 |  | Down |
| *Glucose* | 4.72E-02 |  | Up |
| *Unidentified metabolite* | 2.55E-02 |  | Down |
| *1-Methylnicotinamide.1* | 2.17E-03 |  | Down |
| *Lactose.2* | 3.18E-02 |  | Down |
| *Lactose.3* | 1.88E-03 |  | Down |
| *Unidentified metabolite* | 1.15E-03 |  | Down |
| *Hydroxyacetone* | 4.48E-02 |  | Down |
| *Unidentified metabolite* | 4.09E-03 |  | Down |
| *1-Methylnicotinamide.2* | 1.71E-02 |  | Down |
| *Tartrate* | 3.36E-02 |  | Down |
| *Adenosine* | 4.84E-02 |  | Down |
| *Sucrose.1* | 1.66E-02 |  | Down |
| *Sucrose.2* | 7.93E-03 |  | Down |
| *o-Hydroxyphenylacetic acid, Caffeine.3* | 3.18E-02 |  | Up |
| *Myoinositol, Phenylacetic acid* | 4.84E-02 |  | Up |
| *Malonic acid* | 4.48E-02 |  | Down |
| *3-Phenylpropionic acid.1* | 3.69E-03 |  | Down |
| *Methylguanidine* | 4.15E-02 |  | Down |
| *Levulinic acid* | 1.23E-02 |  | Down |
| *Homocysteine* | 9.30E-03 |  | Down |
| *3-Aminoisobutanoic acid.1* | 4.53E-03 |  | Down |
| *3-Aminoisobutanoic acid.2* | 3.66E-04 |  | Down |
| *3-Aminoisobutanoic acid.4, Methylamine* | 4.26E-02 |  | Down |
| *3-Aminoisobutanoic acid.3* | 2.97E-04 |  | Down |
| *Glutathione* | 1.39E-02 |  | Down |
| *Homoserine* | 4.48E-02 |  | Down |
| *Threonine, Lactic acid* | 1.71E-02 |  | Up |
| *Unidentified metabolite* | 1.81E-02 |  | Down |
| *Fucose* | 4.60E-02 |  | Down |
| *Ethanol.1* | 4.97E-02 |  | Down |
| *Ethanol.2* | 4.84E-02 |  | Down |
| *Methylsuccinic acid, 3-Hydroxyisobutyric acid* | 4.15E-02 |  | Up |
| *2-Octenoic acid.4, Capric acid, 2-Hydroxyisovaleric acid* | 1.39E-02 |  | Down |
| *Unidentified metabolite* | 2.99E-03 |  | Down |
| *Unidentified metabolite* | 2.55E-02 |  | Down |
