# Supplementary material for: Metabolic dysfunction in pregnancy: Fingerprinting the maternal metabolome using proton nuclear magnetic resonance spectroscopy
Source: Endocrinol Diabetes Metab. 2020 Nov 18;4(1):e00201. doi: 10.1002/edm2.201 (PMC7831222; doi:10.1002/edm2.201)
Supplement: Supplementary file 3 — Table S3 [file EDM2-4-e00201-s003.docx]

**Supplementary Table 3.** P-values of urinary metabolites found to be significant in separation of GDM and obese groups in either a Mann-Whitney U test, the Variable Importance Analysis based on random Variable Combination (VIAVC), or both. Regulation is shown for the metabolite levels in the GDM group with respect to the obese group. Metabolites for which more than one NMR resonance peak was identified as significant are represented as metabolite.1, metabolite.2, … metabolite.n.

| Metabolite | Mann Whitney U Test | VIAVC p-value | Regulation |
| --- | --- | --- | --- |
| *Creatine, Caffeine.1* | 1.80E-02 | 4.04E-241 | Up |
| *Sarcosine, Dimethylamine* | - | 2.81E-96 | Down |
| *Maltose, Sucrose* | - | 5.64E-64 | Up |
| *Methionine.2* | 2.86E-03 | 2.91E-55 | Down |
| *Methionine.1* | 2.43E-03 | 6.08E-52 | Down |
| *Leucine* | - | 1.27E-51 | Up |
| *Ethanolamine* | - | 6.79E-50 | Up |
| *Methylsuccinic acid.2, 2-Methylglutaric acid.2, 3-Hydroxyisobutyric acid.2, Isobutyric acid.2* | 2.06E-03 | 9.64E-48 | Up |
| *Alpha-Aminoadipic acid* | 1.08E-02 | 5.46E-44 | Down |
| *3-Hydroxyisovaleric acid, Pyruvic acid* | - | 2.96E-40 | Up |
| *Methionine.3, Methylamine* | 1.11E-02 | 1.15E-31 | Down |
| *Methylsuccinic acid.1, 2-Methylglutaric acid.1, 3-Hydroxyisobutyric acid.1, Isobutyric acid.1* | 3.35E-03 | 4.62E-31 | Up |
| *N-Acetylglutamine.1* | - | 2.39E-20 | Up |
| *1-Methylnicotinamide* | - | 1.34E-19 | Up |
| *Citric acid* | - | 2.27E-18 | Up |
| *Dimethylglycine.2, N-Methylhydantoin* | 1.53E-03 | 1.95E-14 | Down |
| *Trigonelline.2* | - | 6.61E-13 | Up |
| *Trigonelline.1* | - | 3.48E-12 | Up |
| *Trigonelline.3* | - | 2.30E-09 | Up |
| *2-Hydroxyisovalerate* | 2.66E-02 | - | Down |
| *Alanine* | 4.74E-02 | - | Up |
| *Anserine, 1-Methylhistidine, Dimethylglycine.1* | 1.33E-02 | - | Up |
| *cis-Aconitic acid, Malonic acid* | 2.20E-02 | - | Down |
| *Citraconic acid* | 3.86E-02 | - | Down |
| *Creatinine.1* | 2.15E-03 | - | Down |
| *Creatinine.2* | 1.42E-02 | - | Down |
| *Ethanol, Fucose.3* | 1.28E-02 | - | Down |
| *Fucose.1* | 3.52E-02 | - | Down |
| *Fucose.2* | 4.74E-02 | - | Down |
| *Fumaric acid* | 3.35E-03 | - | Up |
| *Glucuronic acid.1, Glucose.1* | 1.99E-02 | - | Up |
| *Glycine, Glucose.2, Glucuronic acid.2, Fructose.2* | 3.63E-03 | - | Up |
| *Histamine, Glucuronic acid.4, Xylose* | 2.93E-02 | - | Up |
| *Lactic acid, 5-Aminolevulinic acid, Fructose.1* | 4.09E-02 | - | Up |
| *Levulinic acid, Acetone* | 4.74E-02 | - | Down |
| *Methylguanidine* | 3.11E-02 | - | Down |
| *N-Acetylglutamine.2* | 6.46E-03 | - | Down |
| *Phenylacetic acid, myoinositol, Glucuronic acid.3, Caffeine.2* | 3.52E-02 | - | Up |
| *Phenylalanine, Threonic acid* | 4.47E-02 | - | Up |
| *Threonine* | 8.06E-03 | - | Up |
| Trimethylamine | 9.66E-03 | - | Down |
